# Emodin Combined with Nanosilver Inhibited Sepsis by Anti-inflammatory Protection

**DOI:** 10.3389/fphar.2016.00536

**Published:** 2017-01-10

**Authors:** Hong Li, Tian Yang, Hong Zhou, Juan Du, Bo Zhu, Zhongmin Sun

**Affiliations:** Department of Respiratory and Critical Care Medicine, The First Affiliated Hospital of Xi'an Jiaotong UniversityXi'an, China

**Keywords:** sepsis, endothelial cells, emodin, nanosilver, NF-κB, p38 MAPK

## Abstract

**Background:** Emodin is the main active component of rhubarb, which has demonstrated many beneficial effects against inflammation. Nanosilver is an effective antimicrobial agent. The present study was designed to observe the effects of Emodin combined with silver nanoparticles (E/S) on sepsis protection and related mechanism.

**Methods:** E/S was prepared by loading different concentrations of Emodin on nanosilver and cytotoxicity of E/S were determined by suphorhodamine B assays. Anti-microbial activities of E/S were assayed by direct interaction with various common pathogens and anti-adhesive activites of E/S on leukocytes with endothelial cells were assayed by biochemical analysis. Next, inflammatory cell enumeration, inflammatory mediators in bronchoalveolar lavage fluid (BALF) and endothelial cell function were analyzed on a clinically relevant model of sepsis induced by cecal ligation and puncture (CLP) after E/S administration. The effects of E/S on NF-κB and p38 were also examined by western blot.

**Results:** E/S exhibited little cytotoxicity action on endothelial cells and significant inhibitory activities against all tested common microorganisms and adherence between leukocyte and endothelial cells. E/S induced anti-sepsis protection mainly mediated by inhibition of inflammatory cells infiltration, down-regulation of TNF-alpha, IL-8 and lactic dehydrogenase (LDH), and inhibition of NF-κB and p38 pathways in mice 24 h post-CLP.

**Conclusion:** Our data suggest that E/S has strong anti-sepsis effects, which was related with anti-inflammatory protection and thereby promote survival following sepsis challenge.

## Introduction

Severe sepsis and septic shock are common fatal diseases with an extremely high morbidity rate for overwhelming lung inflammation in intensive care unit (Mikkelsen et al., 2013) and management of patients relies mainly on early, right and rapid therapeutic measures including administration of appropriate antibiotics, control of infection source, and resuscitation with intravenous fluids and vasoactive drugs when needed (Cohen et al., 2015). Accumulating evidence suggests that a large number of pathogens and subsequent the production of a large quantity of inflammatory mediators such as tumor necrosis factor-α (TNF-α), interleukin-8 (IL-8), reactive oxygen species (ROS) and nitric oxide (NO) etc., mainly through nuclear factor-kappa B (NF-κB) and mitogen-activated protein kinase (MAPK) pathways, gets involved in the pathogenesis of sepsis (Carneiro et al., 2013; Liang et al., 2014). On one hand, a large number of pathogens enter the blood stream, release a large number of pathogenic substances and activate serious systemic inflammatory response. On the other hand, activation of inflammatory signaling resulted in increasing adhesion molecules expressions, including intercellular adhesion molecule (ICAM)-1 and vascular cell adhesion molecule (VCAM)-1, which play significant roles in migration and cell adhesion of various inflammatory cells and further damage to epithelial and endothelial cells in the lung (Rajasingh et al., 2006; Zhang et al., 2006). Thus, Sepsis results from the combined effect of a virulent infection and powerful host response to the infection. Although great efforts has been made in the inhibition of exaggerated inflammation and promotion of endothelial protection, the treatments have still been very limited and cause high mortality rates worldwide.

Modern pharmacology has demonstrated that the active ingredient Emodin of rhubarb exhibits a series of pharmaceutical activities against inflammation, tumors, atherosclerosis and allergies (Wang et al., 2008, 2012; Xiong et al., 2011). Furthermore, it has been found that the anti-inflammatory effect of emodin results from inhibition of the p38 MAPK and NF-κB signaling pathway (Xue J. et al., 2015). Recently, nanosilver has received considerable attention as antimicrobial agents due to its strong anti-microbial activities (Rai et al., 2015), which is especially beneficial against resistant bacteria such as *E. coli, P. aeruginosa* and *A. baumanii* etc (Wang et al., 2015). However, in light of toxicological research, nanosilver has an adverse effect by induction of oxidation stress, contributing to cytotoxic and genotoxic injuries (Wąsowicz et al., 2011). The value of combined nanosilver with emdoin on sepsis is still unclear. Thus, the purpose of this study was to elucidate whether E/S could ameliorate a clinically relevant sepsis and its corresponding mechanism.

## Materials and methods

### Ethics statement

This study was carried out in strict accordance with the recommendations in the Guide for the Care and Use of Laboratory Animals of the National Institutes of Health of China. The protocol was approved by the Committee on the Ethics of Animal Experiments of the University of Xi'an Jiaotong University (Permit Number: 2015-152). All surgery were performed under sodium pentobarbital anesthesia, and all efforts were made to minimize risks.

### Synthesis of E/S

The nanosilver particles were purchased from Alfa Aesar Biotechnology Ltd. (Tianjin, China) with the mean diameter obtained and evaluated by scanning electron microscope (SEM) was 30 nm. Emodin (molecular weight: 270.24) were obtained from Chengdu Biopurify Phytochemicals Ltd. (Chengdu, China) with a purity of at least 99%. Aliquots of an initial 5 mg/mL emodin solution in MeOH were diluted in culture medium to obtain different concentrations to load in nanosilver.

### Cell toxicity of E/S

Human newborn fetal umbilical veins endothelial cells were supplied by Modern Analysis and Testing Center of Xi'an Jiaotong University and seeded into a 96-well plate at a density of 1 × 10^5^ cells per well and incubated in DMEM (Hyclone, USA) supplemented with 12% FBS (GIBCO, USA) at 37°C in 95% air and 5% CO_2_. Different final concentrations of Emodin (1, 10, 20 or 50 μg/ml), Nanosilver (0.1, 1, 5, or 10 μg/ml) and E/S (1, 10, 20 or 50 μg/ml loaded on 1 μg/ml nanosilver) were added into cultured endothelial cells for 72 h, followed by fixing the cells in 30% of trichloroacetic acid for 2 h at 4°C. After 3 times of washes, cells were exposed to 0.5% suphorhodamine B (SRB) solution for 30 min in dark place and subsequently washed with 1% acetic acid. After drying overnight, Tris-HCl was used to dissolve the SRB-stained cells and absorbance was measured at 540 nm. Data are represented as a percentage of control cells.

### Assessment of antimicrobial activity

Pure cultures of *E. coli* (ATCC 8739), *S. aureus* (ATCC 25923), *P. aeruginosa* (ATCC 27853), *S. aureus* (ATCC 25923), and *A. baumanii* (ATCC 19606) *C. albicans* (ATCC 10231) were obtained from Department of Microbiology of Xi'an Jiaotong University. E/S was added into Luria-Bertani (LB) cultured media containing 105 colony-forming units of above microbes per ml (cfu/ml) to a final concentration of 20 μg Emodin/1 μg nanosilver per ml. The test tubes were incubated at 37°C for 24 h. After incubation, 100 μl of the sample was drawn from each test tube and inhibition of growth was determined by measuring absorbance at 600 nm.

### Assay of inflammatory cell adherence

Endothelial cells (1 × 10^5^ cells/well) treated with or without LPS (20 μg/ml) were grown in 24-well plates. The leukocytes were extracted from blood specimens by using lymphocyte separation medium in 10 cm centrifuge tubes. After the top interface was removed and discarded, the leukocytes-containing pellet was resuspended in PBS. The leukocytes (1 × 10^5^ cells/well) were then added to the endothelial cell cultures to a total volume of 0.25 mL and incubated for 4 h at 37°C. After two washes with PBS, cells were added 0.25 ml of 0.5% cetrimid to extract peroxidase (no peroxidase in endothelial cells). Fifty microliter of supernatant reacted with 150 μl of substrate at room temperature for 30 min. The OD492 nm was determined by a microplate reader. The adherence rate was calculated according to standard curve obtained by a serial dilutions of inflammatory cells.

### Establishment of animal models

Fifty male BALB/c mice (weight range, 20–30 g) were randomly and evenly divided into 5 groups: Control group, CLP group, Emodin+CLP group (20 mg/kg), E/S+CLP (20 mg/1 mg/kg) group and nanosilver+CLP (1 mg/kg) group. Briefly, the mice were anesthetized with 60 mg/kg of pentobarbital sodium, and a 1-cm midline abdominal incision was then made. The cecum was ligated, punctured and then returned to the abdominal cavity according to previous research (Zhu et al., 2015). Control mice were treated with cecal manipulations without ligation and puncture. Ten minutes after the operation, mice in CLP group were intraperitoneally administered with E/S, Emodin or nanosilver and mice in comtrol group were administered same medium without drugs. The animals were given analgesic (Buprenorphine, i.p. 0.1 mg/kg, ACRIS, Germany) twice per day and observed every 4 h and put to death by pentobarbital sodium (i.v. 100 mg/kg).

### Immunohistochemistry for tissue morphology

Lungs were inflated, harvested, fixed in 2% paraformaldehyde, and embedded in paraffin. To visualize the endothelial cells, lung sections were immunostained with polyclonal rabbit anti mouse CD31 antibody (Abcam, Cambridge, MA, USA), which were employed as endothelial markers at a dilution of 1:200. The slides were washed and incubated with biotinylated goat anti rabbit IgG for 1 h and washed again. After washing in PBS, the signal was detected with 3, 3′-diaminobenzidine (Dingguo, China).

### Broncho alveolar lavage fluid (BALF) and cell counts

Twenty-four hours later, mice were sacrificed after anesthesia by pentobarbitone sodium (30 mg/kg i.p.). According to our previous report, BALFs were collected by cannulating the upper part of the trachea, by lavage 3 times with 1.0 mL PBS (pH 7.2). The fluid recovery rate was about 85%. Lavaged samples were kept on ice. BALFs were centrifuged at 1000 × *g* for 5 min at 4°C. The sediment cells were resuspended in 50 μL PBS and stained with Wright-Giemsa staining. Then, total cells, neutrophils, macrophages and lymphocytes were counted double-blindly with a hemocytometer.

### Measurement of media release in BALF

TNF-alpha (R&D Systems, USA) and IL-8 (R&D Systems, USA) production in BALFs were measured by ELISA according to the manufacturer's instructions. Briefly, Total 100 μL supernatants were added into 96-well plate and incubated for 1 h, followed by 100 μL enzyme-linked antibodies incubation for 0.5 h at 37°C. After washed for 3 times with washing buffer, the chromogenic reagent was added and incubated for 0.5 h, followed with 2M H_2_SO_4_ termination reaction. The 450 nm absorbance was determined by a microplate reader. Each sample was repeated three times.

Levels of lactic dehydrogenase (LDH) in the BALFs, which can be used as a marker of cellular lysis, were determined by using an LDH assay kit. LDH concentrations were measured by a microplate reader (Tecan Sunrise, China) at 440 nm.

### Western blot analysis

Lung tissues were disrupted by homogenization on ice and centrifuged at 4°C (10,000 *g*, 30 min), and the supernatants were collected. Equal amounts of protein (80 μg) were separated by 10% SDS-PAGE and transferred to duralose membrane. Membranes were blocked in 5% (w/v) skim milk for 1 h and incubated with rabbit against mouse p-p38 MAPK, p38 MAPK, NF-κB p65 (1:1000; Santa cruz, USA) and β-actin (1:2000; Santa cruz, USA) overnight, respectively. After 3 washes, membranes were incubated with horseradish peroxidase conjugated secondary antibodies (Santa Cruz, USA) and detected by enhanced chemiluminescence (Thermo Scientific, USA). Band intensities were quantified using ImageJ (National Institutes of Health, USA).

### Statistical analysis

Data were expressed as mean ± SD. *t*-test was used to analyze differences between experimental and control groups. Student-Newman-Kleus method was used for multiple pair-wise comparisons. All statistical analyses were carried out using the SPSS version 11.5 for Windows (Chicago, IL, USA). *P* < 0.05 was considered statistically significant.

## Results

### Cytotoxicity of E/S on endothelial cells and its anti-microbial and anti-adhesive activities

**Figure 1:**
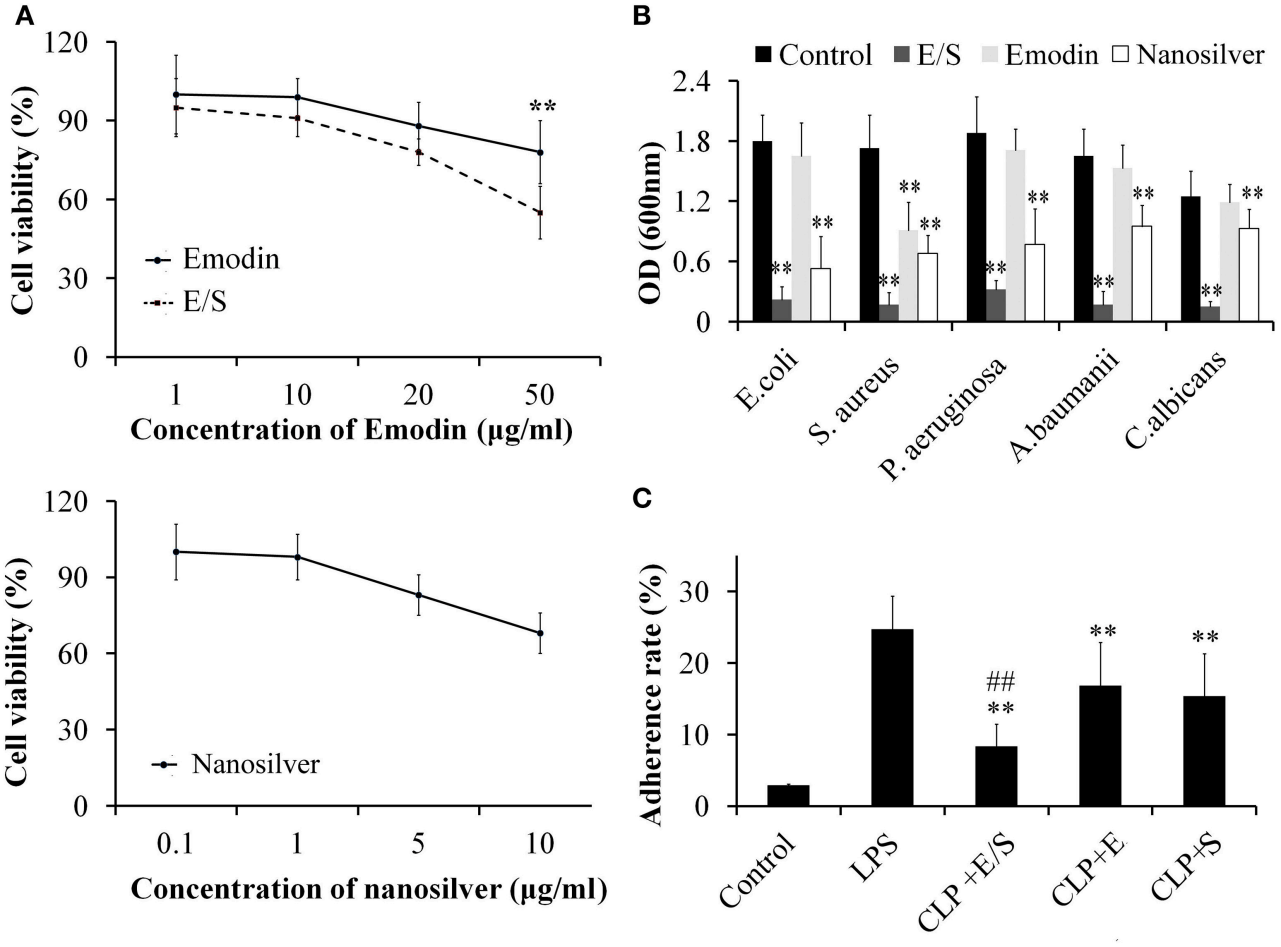
**Cytotoxicity of E/S on endothelial cells** (**A**, ^**^ *p* < 0.01 vs. E/S) and its anti-microbial activities (**B**, ^**^ *p* < 0.01 vs. Control), and anti-adhesive activities (**C**, ^**^ *p* < 0.01 vs. LPS; ^*##*^*p* < 0.01 vs. Emodin or nanosilver) (*n* = 3). The effects of E/S on endothelial cells exhibited little cytotoxicity below 50 μg/ml. And E/S (a final concentration of 20 μg/ml Emodin and 1 μg/ml nanosilver) showed significant inhibitory activities against all tested microorganisms and inflammatory adherence.

Under microscope, the cells exhibited little cytotoxicity action after the administration of different final concentrations of Emodin. E/S exhibited an obvious cytotoxicity at 50 μg Emodin/1 μg nanosilver per mL, while nanosilver exhibited a dose-independent cytotoxicity with an obvious cytotoxicity at 10 μg/mL (Figure 1A). In present study, we choose a final concentration of 20 μg/mL Emodin and1 μg/mL nanosilver to construct the combined administration.

Next, we tested the anti-microbial activities of E/S against common microorganisms in lung infection. Fresh *E. coli, S. aureus, P. aeruginosa, A. baumanii, and C. albicans* were grown in separate LB broths and then E/S (20 μg/ml Emodin and 1 μg/ml nanosilver), Emodin (20 μg/ml) or nanosilver (10 μg/ml) were inoculated in 1 mL LB broth with a concentration of 10^5^ cfu/mL. All the test tubes cultured at 37°C for 24 h. Then, 100 μl of the sample was collected from each test tube and the inhibition of various treatments on bacterial growth was determined by measuring OD600 nm. The analyses showed E/S significantly inhibited all tested microorganisms, while Emodin only inhibited *S. aureus* as shown in Figure 1B. Nanosilver also showed significant inhibition against all tested microorganisms, however, E/S had much stronger inhibitory effects than nanosilver. So antimicrobial effect of E/S is greater than when they (E and S) apply separately.

Next, we measured the effects of E/S on inflammatory adherence. We found that the adherence of leukocytes to endothelial cells was low in control cells. After treatment of LPS (20 μg/ml), leukocyte adherence significantly increased 10-folds. E/S, E or nanosilver resulted in significant inhibition of leukocyte adherence to endothelial cells induced by LPS, however, E/S had much stronger inhibitory effects (Figure 1C).

### E/S ameliorates lung inflammation in a sepsis animal model

**Figure 2:**
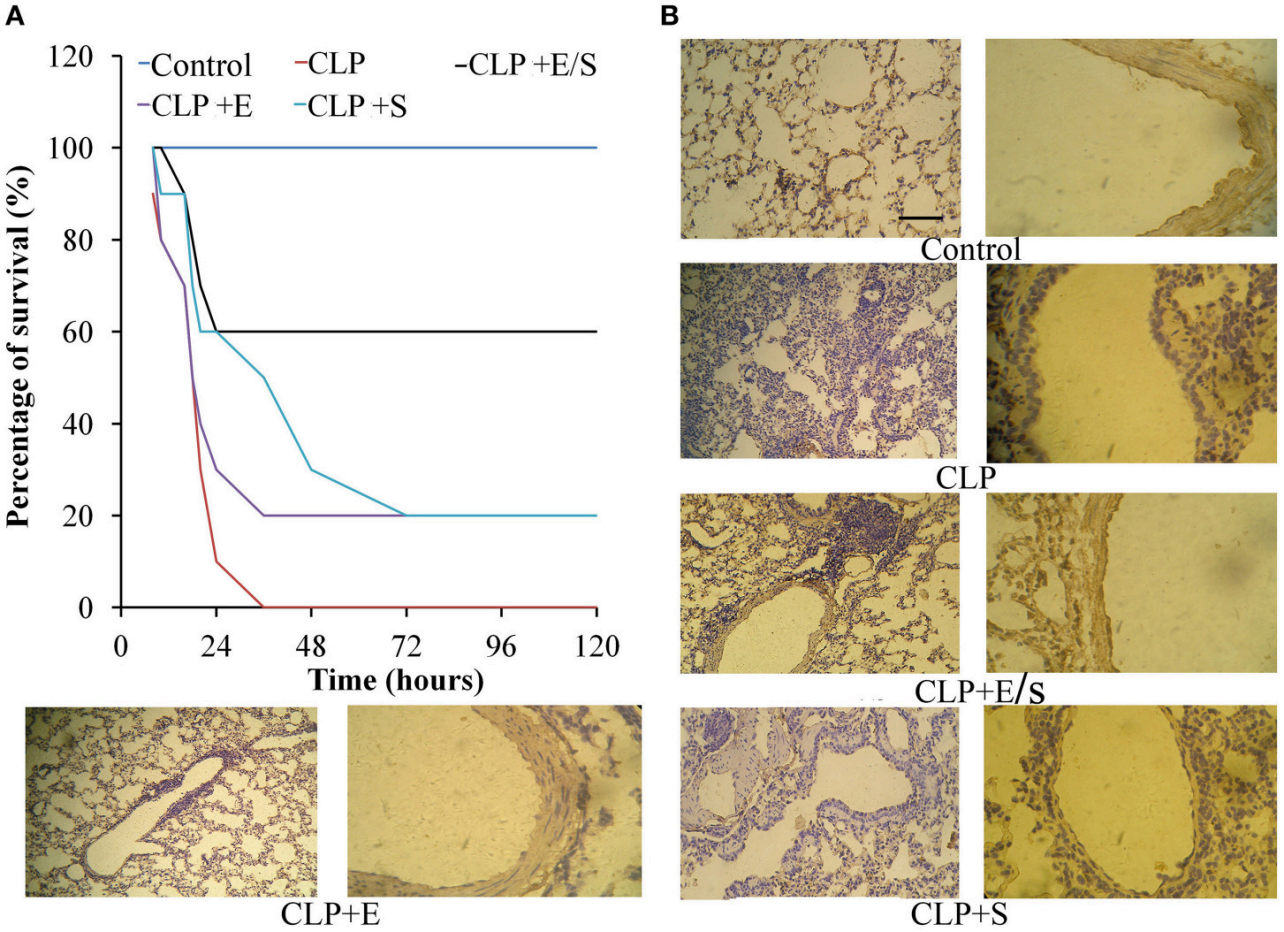
**Effects of E/S on lethal toxicity (A)** and lung inflammation **(B)** in a CLP-induced sepsis model (*n* = 10). Balb/c mice were administered E/S (20 mg/1 mg/kg). Lethality was evaluated within 120 h after CLP challenge. E/S significantly decreased the lethality. Immunohistochemistry of endothelial cells **(B)** revealed that E/S markedly promoted the endothelial staining and reduced the infiltration of inflammatory cells induced by CLP challenge.

All mice in the control group survived. In the CLP-challenge group, the mice died at 10–12 h, and the lethality reached 100% at 36 h. Pretreatment of the mice with E/S (20 mg/1 mg/kg) significantly decreased the lethality to 30% (Figure 2A). To observe the pathological changes of the lung tissues, immunohistochemistry was utilized in our study. As shown in Figure 2B, immunohistochemistry of endothelial cells revealed that the lung tissues from the sepsis group demonstrated significantly pathological alterations, including notable inflammatory cells infiltration, interstitial and intra-alveolar edema and some collapsed alveoli, which were attenuated by E/S or Emodin. Moreover, it was shown that the immune staining of endothelial cells enhanced in E/S group 24 h post-CLP.

**Figure 3:**
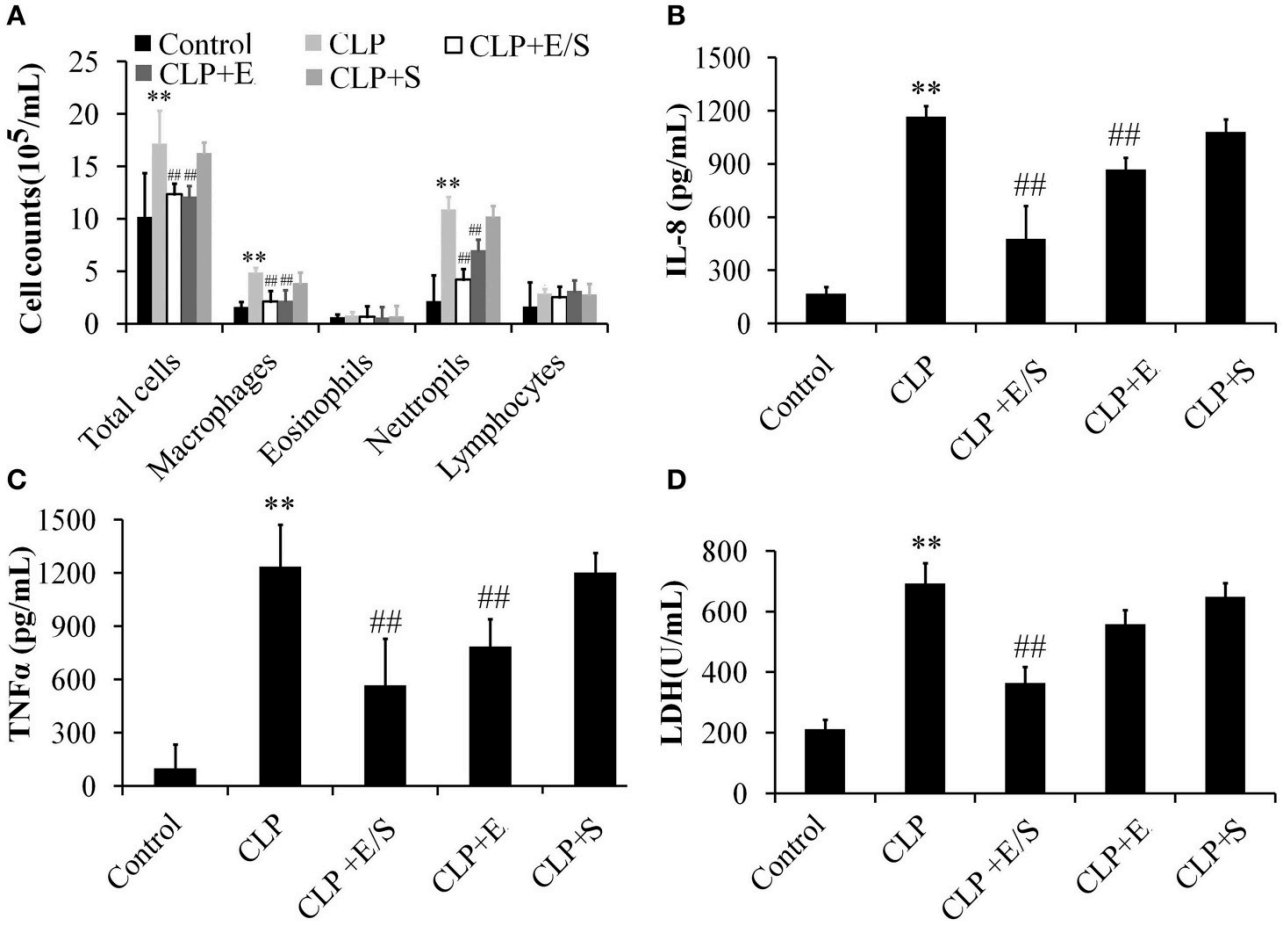
**Effects of E/S on lung functions in CLP-induced sepsis (***n*** = 6)**. Balb/c mice were administered E/S (20 mg/1 mg/kg). The inflammatory cell numbers **(A)**, IL-8 levels **(B)**, TNF-α levels **(C)** and LDH release **(D)** in BALFs increased at 24 h post-CLP challenge in a manner that was inhibited by E/S. ^**^ *p* < 0.01 vs. Control; ^*##*^*p* < 0.01 vs. CLP.

To further evaluate the anti-inflammatory property of E/S, cell counts in BALF were measured in our study. As shown in Figure 3A, the number of total cells, neutrophils and macrophages in BALFs significantly increased in CLP group. E/S or Emodin group largely reduced the number of total cells, neutrophils and macrophages in BALFs. The TNF-α levels (Figure 3B) and IL-8 levels (Figure 3C) in BALFs increased at 24 h post-CLP challenge in a manner that was inhibited by E/S or Emodin. Under the condition of cellular necrosis or apoptosis, cells lose cell membrane stabilization and thereafter release LDH. We next quantified LDH leakage as a parameter of cell membrane integrity. LDH levels increased in BALF which were subjected to sepsis. The addition of E/S led to the decrease of LDH release (Figure 3D).

### E/S inhibited NF-κB and P38 activation

**Figure 4:**
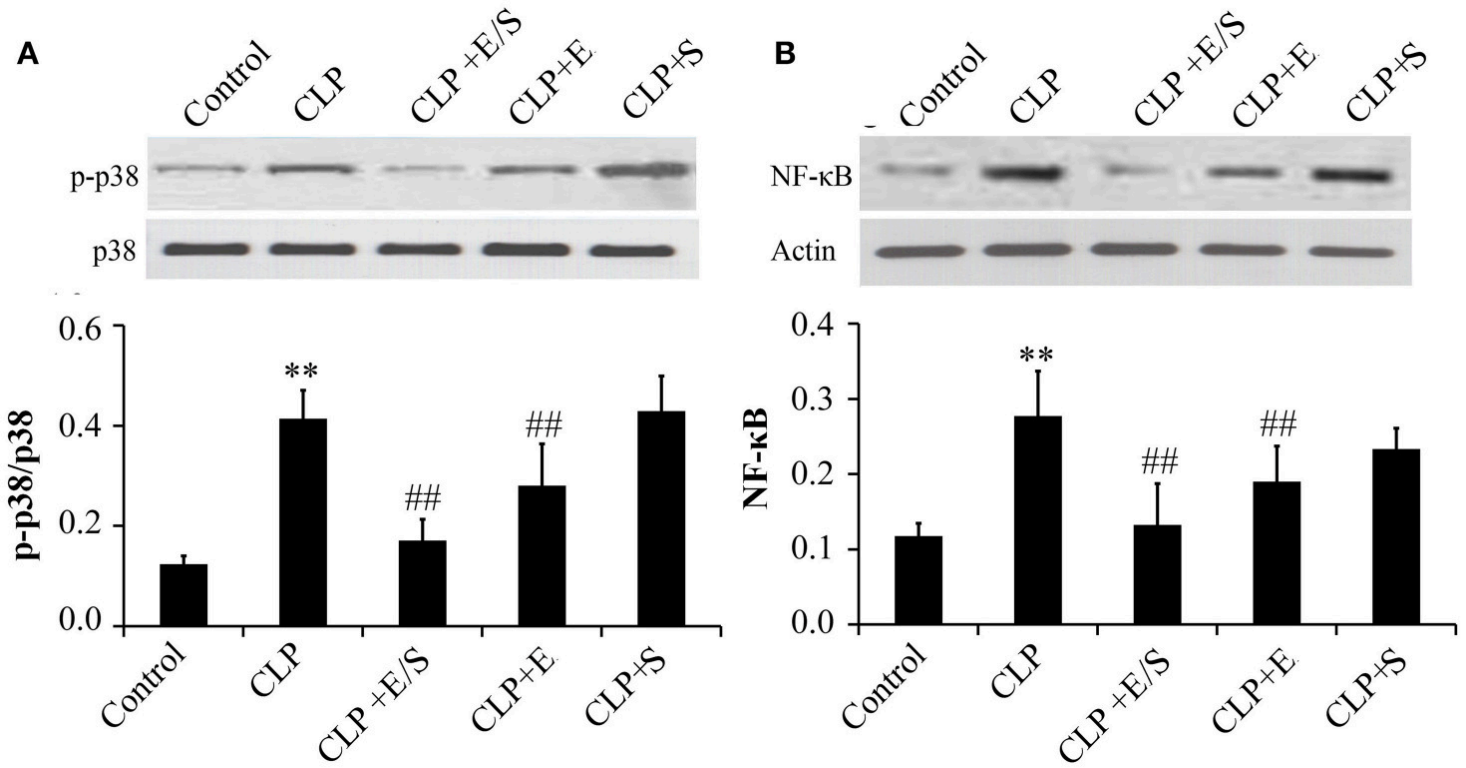
**Effects of E/S on p38 MAPK (A)** and NF-κB **(B)** in CLP-induced sepsis (*n* = 6). Balb/c mice were administered E/S (20 mg/1 mg/kg). p38 MAPK and NF-κB increased at 24 h post-CLP challenge which was inhibited by E/S or Emodin. ^**^p < 0.05 vs. Control; ^*##*^*p* < 0.01 vs. CLP.

Studies have shown that the activation of NF-κB and p38 MAPK triggered the mRNA expression of key inflammatory mediators, including IL-6, IL-8, and TNFα mRNA (Xue J. et al., 2015). Thus, NF-κB and p38 MAPK-induced activation appear essential for inflammatory process. To confirm the protective effect of E/S, western analysis was performed on lung tissues from sepsis animal models. The data showed that the treatment with E/S or Emodin inhibited NF-κB and p38 MAPK signaling in CLP-induced sepsis (Figures 4A,B).

## Discussion

Sepsis is a systemic inflammatory response to infection, in which there is fever or hypothermia, tachycardia, tachypnea, and evidence of inadequate blood flow to internal organs. Severe sepsis results in a high mortality rate in critically ill patients. Over-activated inflammatory cells, mainly neutrophils and macrophages released a variety of inflammatory mediators that could directly and indirectly lead to alveolar epithelium and microvascular endothelium injuries in lung during pathogenesis of sepsis (Lee et al., 2015). Despite substantial progress being made in understanding of this disease, the available treatments are still limited in clinical practice and are mainly supportive treatment, such as mechanical ventilation and nutritional support, and etiological treatment.

Emodin, the bio-active compound of *Radix rhizoma* Rhei shows multiple pharmacological values, including anti-inflammatory, anti-fibrosis, anti-tumor proliferation, anti-atherosclerotic and immunosuppressive effects (Liu et al., 2011; Pang et al., 2015; Xue, W. H. et al., 2015). Nanosilver, shows robust effects against various microorganisms, at the same time, it causes intracellular reactive oxygen species (ROS) generation, DNA damage and apoptosis with a dose-dependent response over 1μg/ml (Orta-García et al., 2015), which limits the utilization of nanosilver *in vivo*. In this study, we found that combination Emodin with low concentration of nanosilver (1μg/ml) had the best anti-microbial and anti-adhesive activities. Anti-adhesive activities of E/S further prevent inflammation by inhibiting migration and adhesion of various inflammatory cells and subsequent damage to endothelial and epithelial cells.

In our study, typical and severe pathological alterations were found in a CLP-induced sepsis model, including notable inflammatory cell infiltration, interstitial and intra-alveolar edema and some collapsed alveoli. CLP also significantly increased total cells, neutrophils and macrophages in BALFs in mice. And these typical pathological changes were remarkably improved by E/S treatment or Emodin. Although Emodin showed protective effects on lung injury, which was consistent with the results of Yin's report (Yin et al., 2016), E/S showed a much stronger inhibition than Emodin, indicating that E/S can strength the effects of Emodin or nanosilver and weak the toxicity of nanosilver.

Our results also suggest that the anti-inflammatory property of E/S possibly results from the inactivation of NF-κB and p38 MAPK signaling pathway, which is consistent with Xue's report (Xue J. et al., 2015). LPS, a major outer membrane component of Gram-negative bacteria, is the main contributing factor to the development of inflammation by binding with its receptor TLR4, which activates several signaling cascade including the mitogen activated protein kinases (MAPKs) such as ERK, p38, and JNK kinases and NF-κB. p38 MAPK and (or) NF-κB inhibitors were reported to inhibit LPS-induced sepsis effectively through multiple pathway (Koch et al., 2012; Chen et al., 2016). Emodin was reported to protect against concanavalin A-induced hepatitis in mice through inhibiting activation of the p38 MAPK-NF-κB signaling pathway (Xue J. et al., 2015), indicating that anti-sepsis effects of Emodin may be closely related with p38 MAPK-NF-κB signaling pathway. There are two potential limitations in current study. First, we revealed protective effects of E/S on sepsis in mice. However, the *in vivo* toxicity and pharmacokinetics of E/S are poorly. Further detailed pharmacokinetics study of E/S in anti-sepsis trials may be warranted. Second, although the obvious improvement against sepsis, the detailed molecular mechanism of E/S on sepsis need further study.

## Conclusions

In conclusion, the E/S have greater antimicrobial effect than when they apply separately. E/S effectively protected against CLP-induced sepsis *in vitro* and *in vivo*. These beneficial effects may be the result of two activities, first, anti-microbial activities and second, suppression of inflammatory activation partly through NF-κB and p38 MAPK pathway, therefore represent potential targets of E/S for the prevention and treatment of sepsis.

## Author contributions

ZS, Conceived the experiments; HL, TY, and HZ, Performed the experiments; JD and BZ analyzed the data; HL wrote the manuscript.

### Conflict of interest statement

The authors declare that the research was conducted in the absence of any commercial or financial relationships that could be construed as a potential conflict of interest.
